# Supplementary material for: Tiron Has Negative Effects on Osteogenic Differentiation via Mitochondrial Dysfunction in Human Periosteum-Derived Cells
Source: Int J Mol Sci. 2022 Nov 14;23(22):14040. doi: 10.3390/ijms232214040 (PMC9693013; doi:10.3390/ijms232214040)
Supplement: Supplementary file 1 [file ijms-23-14040-s001.zip › ijms-1993784-supplementary.pdf]

Supplementary Figure

Tiron Has Negative Effects on Osteogenic Differentiation via Mitochondrial Dysfunction in Human Periosteum-Derived Cells

Jin-Ho Park [1,2], Eun-Byeol Koh [1,2], Young-Jin Seo [1,2], Hye-Seong Oh [1,2], Ju-Yeong Won [1,2], Sun-Chul Hwang [3] and June-Ho Byun [1,2,*]

[1] Department of Oral and Maxillofacial Surgery, Institute of Health Sciences, School of Medicine, Gyeongsang National University, Gyeongsang National University Hospital, Jinju 52727, Korea
[2] Department of Convergence Medical Science, Gyeongsang National University, Jinju 52828, Korea
[3] Department of Orthopaedic Surgery, Institute of Health Sciences, School of Medicine, Gyeongsang National University, Jinju 52828, Korea
* Correspondence: surbyun@gnu.ac.kr

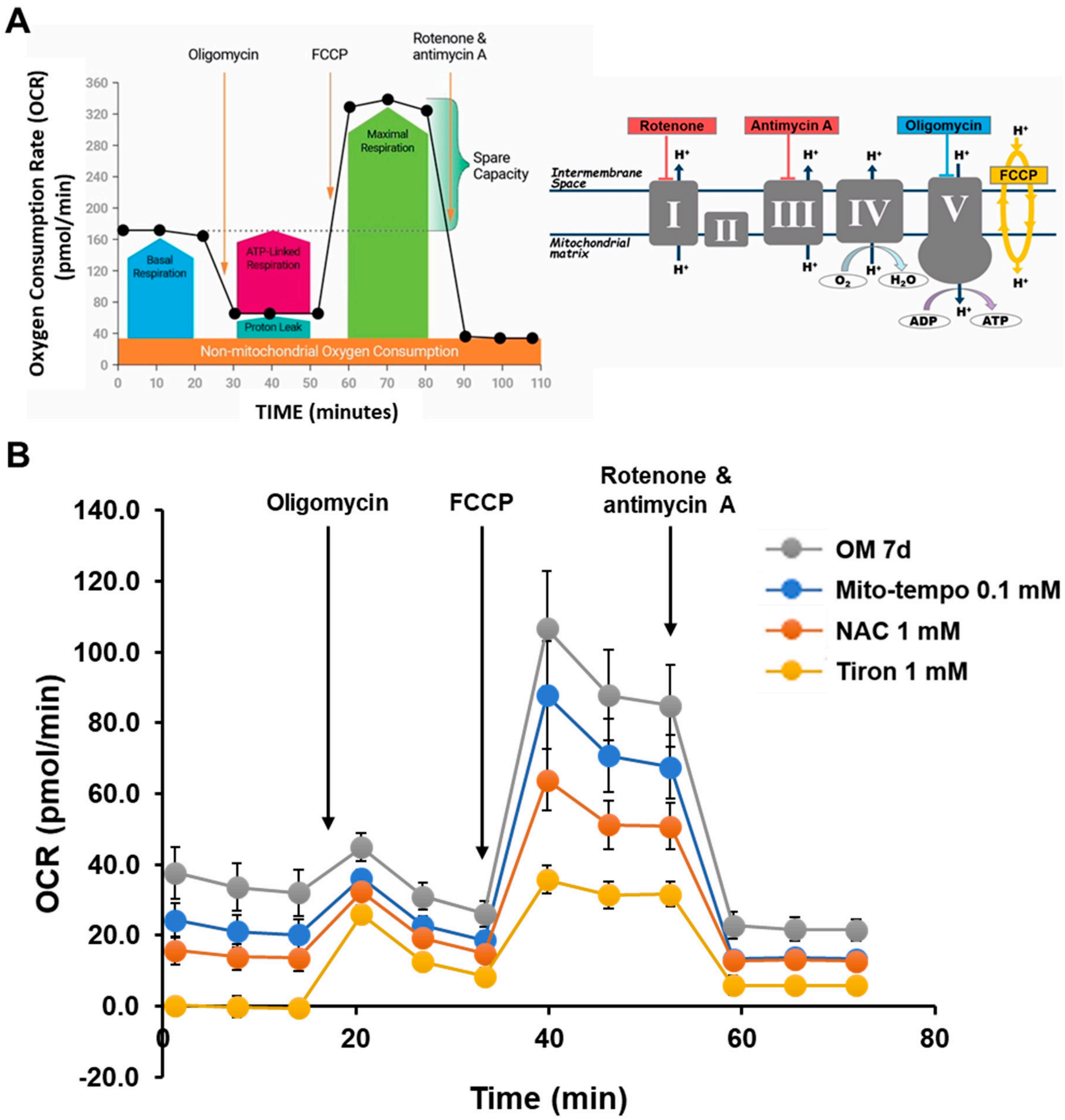

Figure S1. Real-time data of metabolism analysis in Seehorse XF assay. (**A**) Schematic diagram of real-time cell metabolism analysis. (**B**) Real-time cellular mitochondrial stress measurement data of hPDCs at the 1 week of osteogenic differentiation according to each antioxidant treatment.
